# Factors influencing timely diagnosis in neurolymphomatosis

**DOI:** 10.1007/s11060-024-04792-2

**Published:** 2024-08-08

**Authors:** Sofia Doubrovinskaia, Antonia Egert, Philipp Karschnia, Georg T. Scheffler, Benjamin-Leon Traub, Daniela Galluzzo, Anita Huttner, Robert K. Fulbright, Joachim M. Baehring, Leon D. Kaulen

**Affiliations:** 1 Department of Neurology, Yale School of Medicine, New Haven, USA; 2 Department of Neurology, Heidelberg University Hospital, Heidelberg University, Heidelberg, Germany; 3 Department of Neurosurgery, Ludwig Maximilian University (LMU), Munich, Germany; 4 Clinical Cooperation Unit Neuro-Oncology, German Consortium for Translational Cancer Research (DKTK), German Cancer Research Center (DKFZ), Heidelberg, Germany; 5 Department of Pathology, Yale School of Medicine, New Haven, USA; 6 Department of Radiology and Biomedical Imaging, Yale School of Medicine, New Haven, USA; 7 Department of Neurosurgery, Yale School of Medicine, New Haven, USA

## Abstract

**Background:**

Neurolymphomatosis refers to infiltration of the peripheral nervous system (PNS) by non-Hodgkin lymphoma (NHL). Diagnostic intervals in neurolymphomatosis and factors delaying diagnosis have not been evaluated. We therefore aimed to analyze diagnostic intervals in a large cohort.

**Methods:**

The quality control database at Yale Cancer Center, Section of Neuro-Oncology, was searched for neurolymphomatosis cases diagnosed between 2001 and 2021. Univariate analyses were performed to identify parameters influencing diagnostic intervals.

**Results:**

We identified 22 neurolymphomatosis cases including 7 with primary and 15 with secondary disease, which occurred a median (range: 4–144) of 16 months after initial NHL diagnosis. Patients typically presented with painful polyneuropathy (73%), that was asymmetrical and rapidly progressive. Diagnosis was based on PNS biopsy (50%) or integration of neuroimaging findings (50%) with NHL history and diagnostic cerebrospinal fluid examinations. Median interval from symptom onset to diagnosis was 3 months (range: 1–12). Secondary neurolymphomatosis compared to primary disease (median 2 vs. 6 months, *p* = 0.02), and cases with rapidly-progressive asymmetrical neuropathy as opposed to other presentations (median 2 vs. 6 months; *p* < 0.001) were diagnosed earlier. Upfront conventional CT compared to other modalities (median 2 vs. 5 months *p* = 0.04) and nerve root localization as opposed to other disease sites (median 1.5 vs. 4 months; *p* = 0.04) delayed diagnosis.

**Conclusions:**

NL type and localization, neuropathy course and distribution, and imaging modality selected for initial evaluation influence diagnostic intervals in neurolymphomatosis. Knowledge of this rare entity is critical for early suspicion, and diagnosis.

**Supplementary Information:**

The online version contains supplementary material available at 10.1007/s11060-024-04792-2.

## Introduction

Neurolymphomatosis (NL) refers to the invasion of the peripheral nervous system (PNS) by non-Hodgkin lymphoma (NHL) [1–5]. It predominantly results from dissemination of NHL to the PNS (secondary NL) [1–4]. However, in rare cases NL is found at first diagnosis of hematologic malignancy (primary NL) [1–4]. Although the precise incidence is yet to be determined, clinical practice suggests NL constitutes a rare sequel of NHL, noted in < 1% of cases *ante mortem*.

The clinical presentation of NL is diverse and mainly includes four previously described patterns: painful involvement of nerves or nerve roots, cranial neuropathy with or without pain, painless involvement of peripheral nerves and painful or painless involvement of a single peripheral nerve [1]. Clinical presentation typically reflects disease location within the PNS and may hence mimic various other mostly non-neoplastic etiologies (e.g., radiculopathy following disc herniation, diabetic or chemotherapy-induced peripheral neuropathy, chronic inflammatory demyelinating polyneuropathy) [6–8]. Its rarity and the diverse clinical presentation complicate prompt NL diagnosis. As a result, substantial delays may occur during diagnostic work-up. However, diagnostic intervals and factors facilitating or delaying diagnosis have not been thoroughly evaluated.

In the present study, we aimed to summarize the diagnostic work-up of 22 NL cases, diagnosed over a 20-year period at a tertiary referral center. Our primary goal was to identify factors influencing intervals from symptom onset to definitive NL diagnosis considering clinical, neuroradiological and pathological characteristics. Results may guide physicians in appropriate diagnostic work-up and early diagnosis of NL.

## Materials and methods

For this retrospective study, the Quality Control Database of the Section of Neuro-Oncology at Yale Cancer Center was screened for cases of definitive NL, defined as PNS infiltration by NHL, diagnosed *ante- or postmortem* at Yale Brain Tumor Center over a 20-year period (2001–2021). We excluded cases with bulky disease entrapping the PNS and isolated cerebrospinal fluid (CSF)-positive leptomeningeal disease involving cranial nerves or cauda equina following previous work [2]. When dissemination to the PNS was found at the time of first diagnosis of hematolymphoid malignancy, cases were defined as primary NL. Patients with previously diagnosed NHL, which spread to the PNS during the disease course, were considered secondary NL. Pathological diagnosis from PNS biopsy specimen or cerebrospinal fluid (CSF) was mandated in all cases of primary NL. For secondary NL, where NHL had previously been pathologically confirmed, diagnosis could be either tissue- (e.g., nerve biopsy) or imaging-based (e.g. PNS infiltration on follow-up neuroimaging) [2].

Onset and course of symptoms were extracted from patient history. The date of NL diagnosis was defined as date of pathological diagnosis. In cases of secondary NL with imaging-based diagnosis, radiology report date was registered as the date of diagnosis. To estimate diagnostic intervals, the time from onset of symptoms to diagnosis of NL was measured in months. Clinical, imaging and neuropathological data were retrospectively extracted via chart review. Staging is reported according to the Ann Arbor classification given staging systems currently used, i.e. the Lugano classification, had not been available at the time of diagnosis in several included cases [9, 10]. Neuropathy was defined as rapidly progressive when clinical worsening within four weeks was found. Cases with progression occurring over > 4 weeks were defined as slowly progressive. In secondary NL cases histopathological diagnosis was inferred from previous histopathological examinations outside the nervous system. Disease location was determined based on neuroimaging.

Cohort characteristics were summarized using descriptive statistics (e.g., median, ranges). Primary endpoint was the interval from symptom onset to definitive NL diagnosis. Cox proportional hazard regression analysis and log-rank test were used to assess factors influencing this interval on univariate analysis. Findings were considered statistically significant when *p*-values were < 0.05. Statistics were performed using SPSS Version 27 (IBM), and Prism version 10 (GraphPad Prism).

This study was approved by the Yale University Institutional Review Board. Informed consent was waived based on United States federal regulation 45 CFR 46.116 (d). One case from this series was previously published as a case study [4]. Anonymized clinical data is available from the corresponding authors upon reasonable request by any qualified researcher.

## Results

A total of 22 NL cases diagnosed between 2001 and 2021 at Yale Cancer Center were retrospectively identified. Patients were diagnosed at a median age of 56 years (range: 34–79) and male-to-female ratio was 1.75:1. The cohort included 7 (32%) and 15 (68%) cases of primary and secondary NL, respectively. Among primary NL, three cases (3/7, 43%) exhibited additional NHL lesions outside the nervous system. Secondary NL was diagnosed a median of 16 months (range: 12–144) after initial NHL (Supplementary Fig. 1A). Of note, most of these patients had previously been diagnosed with advanced stage NHL (Supplementary Fig. 1B) corresponding to Ann Arbor stages III or IV (10/15, 67%). Extranodal disease was common at initial NHL diagnosis and frequently localized to male or female reproductive organs.

### Diagnostic algorithm

**Fig. 1:**
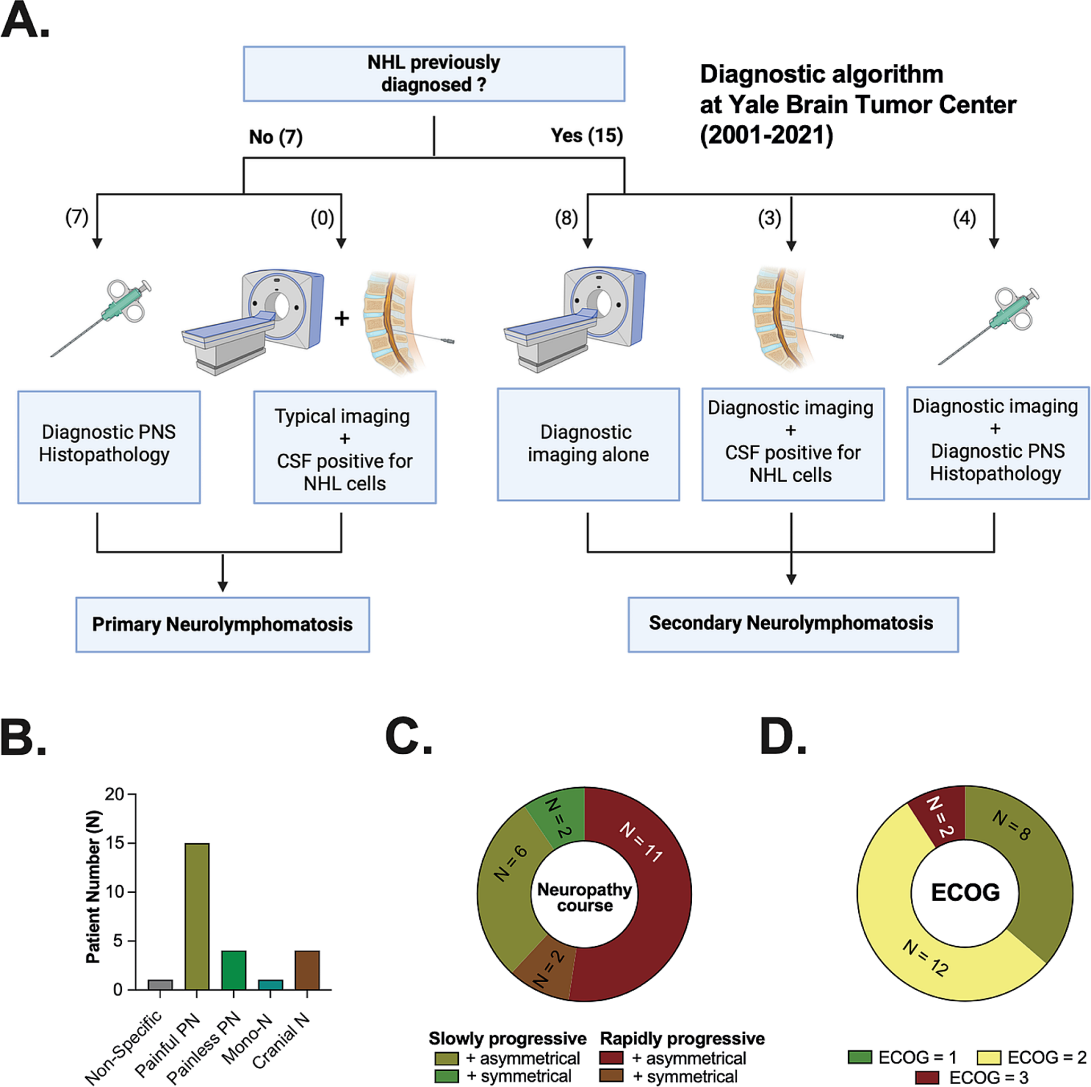
Title: Diagnostic work-up and clinical presentation. Legend: **A**. Diagnostic work-up for neurolymphomatosis (NL) at Yale Brain Tumor Center (YBTC). The number of cases diagnosed using indicated modalities is denoted in parentheses. While primary NL was diagnosed based on histopathology in all cases, secondary NL diagnosis typically relied on neuroimaging and previously diagnosed NHL. This panel was created with BioRender.com **B**. Clinical presentation and **C**. course and distribution of neuropathy are displayed. Most patients presented with painful PN. Neuropathy was typically characterized by an asymmetrical distribution and rapidly progressive course. **D.** Median Eastern Cooperative Oncology Group (ECOG) performance status at diagnosis was 2

Diagnostic work-up in our cohort is summarized in Fig. 1A. All cases were diagnosed *ante mortem*. Diagnosis of primary NL relied on PNS biopsy in all cases (7/7, 100%). Secondary NL was diagnosed based on integration of previous NHL diagnosis with neuroimaging findings. Detection of NHL in the cerebrospinal fluid (CSF) and PNS biopsy further supported the diagnosis in three (3/15, 20%) and four (4/15, 27%) patients, respectively. All performed biopsies were taken from FDG-avid lesions (11/11, 100%) and yielded diagnostic results.

### Clinical presentation

Clinical presentation is summarized in Fig. 1B/C and included painful (16/22, 73%) or painless polyneuropathy (4/22, 18%), and cranial (3/22, 14%) neuropathy. No neurological symptoms were present in one case (1/22, 5%). Distribution of neuropathy was typically asymmetrical with a rapidly progressive course (11/22, 50%, Fig. 1C). However, asymmetrical neuropathies with slower disease progression were also common (6/22, 27%). Eastern Cooperative Oncology Group (ECOG) performance status at diagnosis was poor (ECOG ≥ 2) in the majority of NL cases (14/22, 64%).

### Neuroimaging

**Fig. 2:**
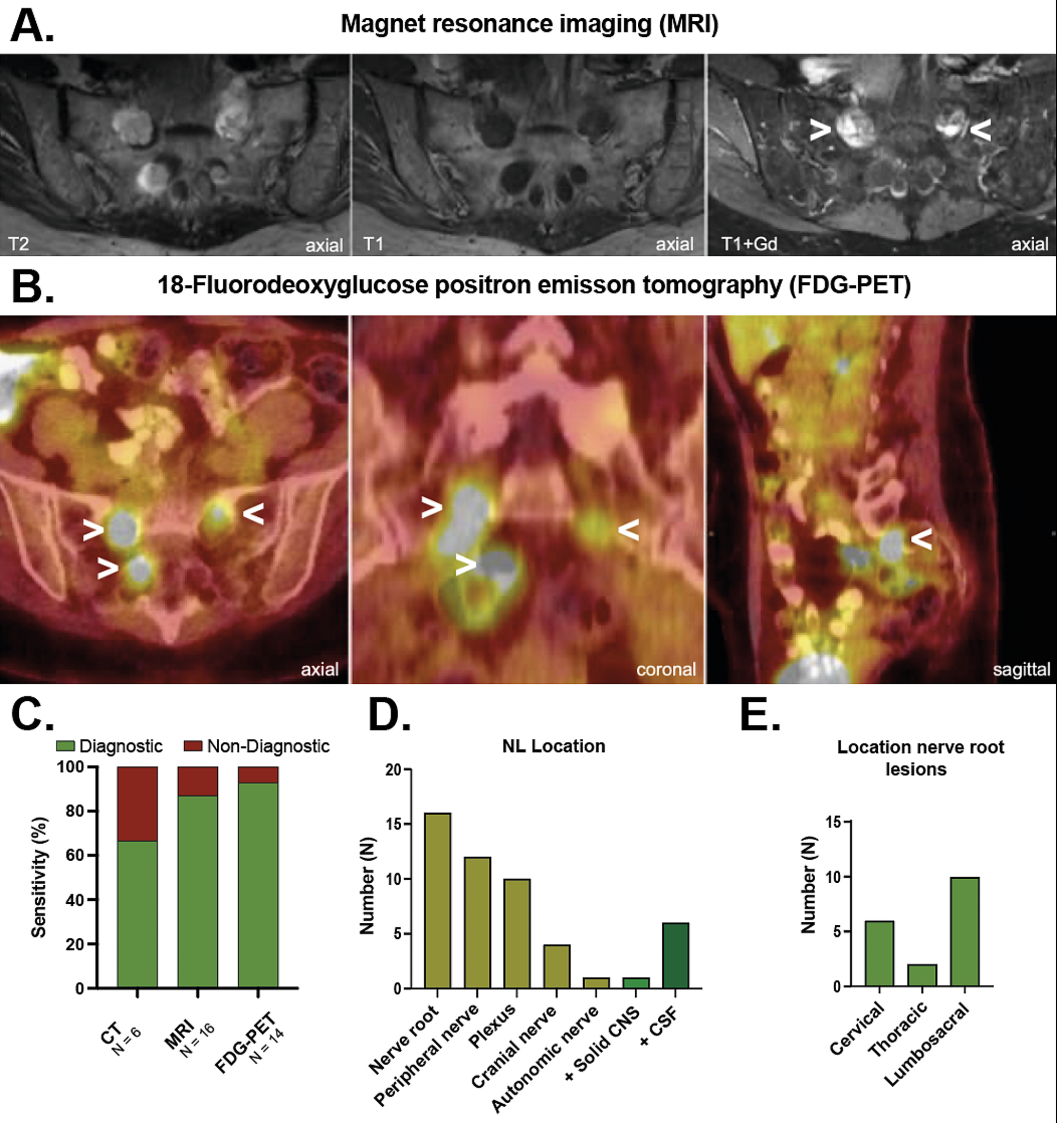
Title: Imaging findings. Legend: MRI (**A.**) and FDG-PET (**B.**) imaging findings are depicted in a case of sacral NL. Sequences and planes are indicated. **A.** Bilateral contrast enhancing S1-nerve root lesions (arrows) were found on MRI. **B.** Lesions display corresponding FDG-avidity (arrows) on FDG-PET. Of note, this patient had additional congenital cystic CSF-filled enlargement (T2- hyperintensities) of sacral nerve root sleeves (Tarlov cysts) unrelated to NL. **C**. Sensitivity of various neuroimaging modalities employed in our cohort is shown. FDG-PET exhibited the highest sensitivity (93%). **(D)** NL location and **(E)** distribution of nerve root lesions are shown. NHL most frequently infiltrated nerve roots and peripheral nerves

Neuroimaging was available in all cases from this study. Characteristic radiological NL findings on MRI (Fig. 2A) and FDG-PET (Fig. 2B) are shown. Typical findings include enlarged structures within the PNS, that enhance after gadolinium contrast agent administration (Fig. 2A) and display FDG-avidity (Fig. 2B). Of note, most NL patients were examined with more than one neuroimaging modality (12/22, 55%). All except one patient were evaluated with at least either MRI or FDG-PET (21/22, 95%). Among these patients, MRI alone, FDG-PET alone or both modalities were used in 7/21, 5/21, and 9/21 cases, respectively. Sensitivity of various neuroradiological examinations in our cohort is depicted in Fig. 2C. Sensitivity of CT, MRI, and FDG-PET was 67% (4/6), 87% (14/16), and 93% (13/14), respectively.

Distribution of NL lesions within the PNS per neuroimaging are presented in Fig. 2D/E. NL predominantly involved nerve roots (16/22, 73%), followed by peripheral nerves (12/22, 55%), and plexus (10/22, 45%). Infiltration of cranial (4/22, 18%) and autonomic nerves (1/22, 5%) was less common (Fig. 2D). Lumbosacral nerve root lesions were more common (10/22, 45%) than infiltration of cervical (6/22, 27%) and thoracic (2/22, 9%) regions (Fig. 2E). Solid masses within the central nervous system were additionally found in one case (1/22, 5%).

### Electromyographic/nerve conduction studies and CSF examinations

**Fig. 3:**
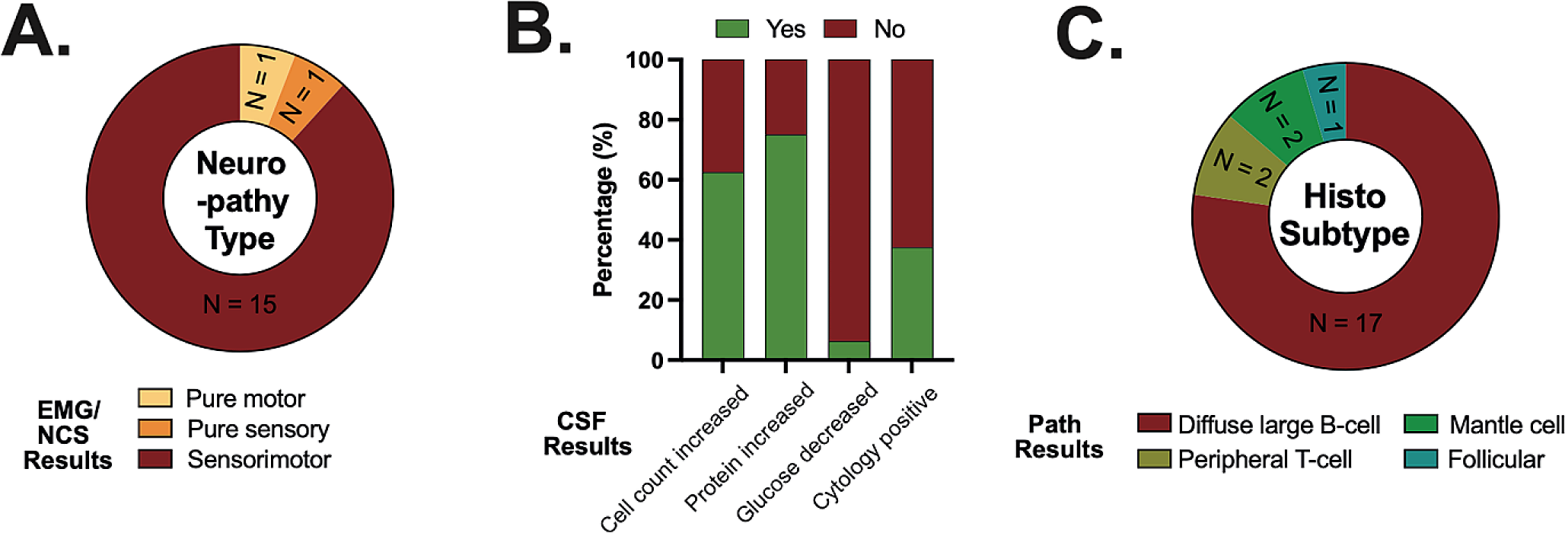
Title: Electrophysiological, cerebrospinal fluid and histopathological findings. **Legend: (A)** Electromyography/ nerve conduction study (EMG/ NCS) revealed sensorimotor neuropathy in most patients. **(B)** Cerebrospinal fluid (CSF) was remarkable for increased cell counts and increased protein in most cases. Glucose levels were usually within the normal range. Malignant cells were found in 38% of cases. **(C)** Diffuse large B-cell lymphoma (DLBCL) was the most common histological NHL subtype found in NL cases

Results from electromyographic and nerve conduction studies of symptomatic PNS structures were available in 17 patients and revealed axonal neuropathy in all of them. Most cases were classified as axonal sensorimotor neuropathy (15/17, 88%) whereas pure motor and sensory axonal neuropathy were noted in one case each (1/17, 6%) (Fig. 3A).

Results from CSF examinations are detailed in Fig. 3B. They were remarkable for lymphocytic pleocytosis in most NL patients (10/16, 63%) with a median cell count of 11 cells/µl (range: 4–100 cells/µl). Elevated CSF protein was detected in 12/16 cases (75%). Glucose levels were unremarkable in most NL cases with decreased levels found in one case (1/16, 6%) only. Malignant NHL cells were detected in the CSF of 6/16 NL cases (38%). Of note, conventional cytologic evaluation was negative in one of these cases, but flow cytometry revealed a malignant B-cell population with an abnormal kappa/ lambda ratio.

### Histopathological characteristics

Histopathological examination typically revealed infiltration and subsequent destruction of ganglia or nerve cells by large, pleomorphic lymphoid cells. Histological subtypes associated with NL are depicted in Fig. 3C and mainly included B-cell NHL (20/22, 91%). DLBCL was the predominant histological subtype, accounting for 17/22 (77%) cases. Other histological subtypes in NL included peripheral T-cell lymphoma (2/22, 9%), mantle cell lymphoma (2/22, 9%) and follicular lymphoma (1/22, 5%).

### Diagnostic intervals and influencing factors

**Fig. 4:**
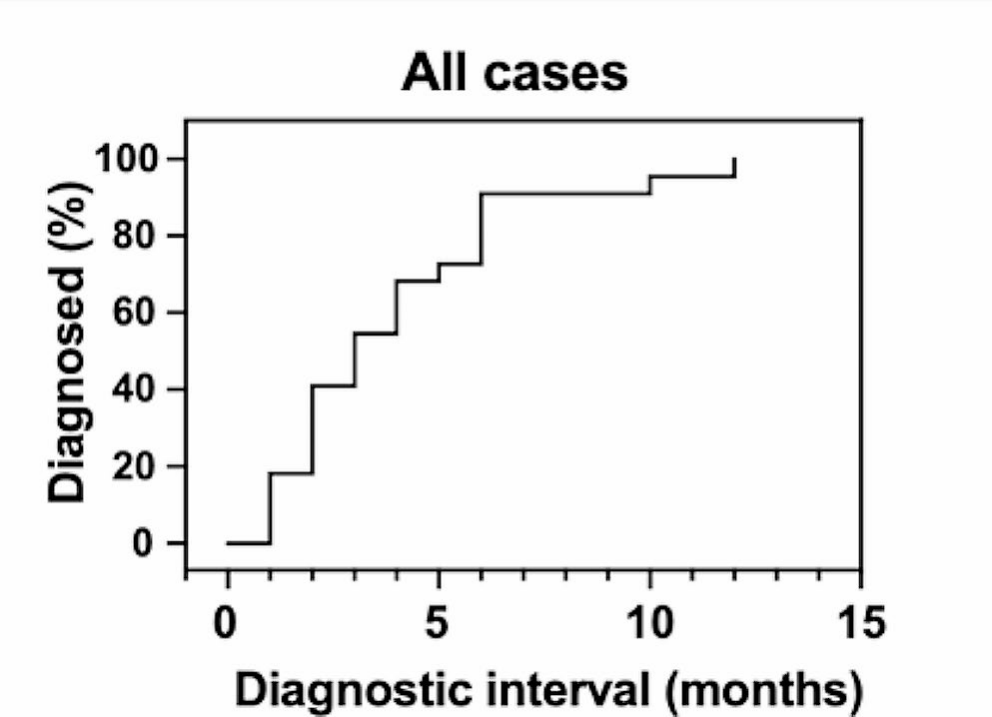
Title: Diagnostic interval in NL. Legend: Intervals from symptom onset to diagnosis are shown for the entire cohort in a reverse Kaplan-Meier curve. NL was diagnosed a median of 3 months after symptom onset

**Fig. 5:**
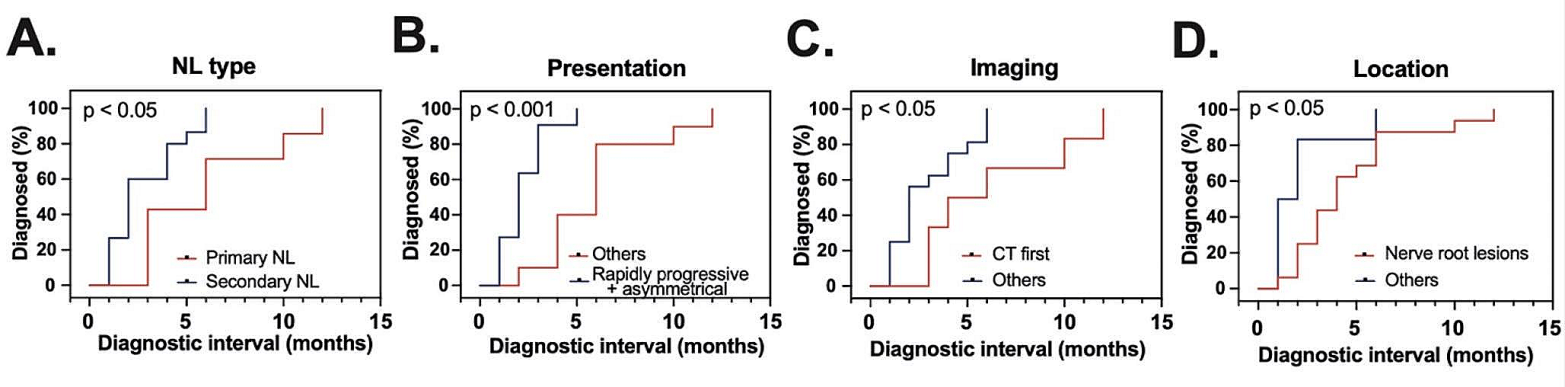
Title: Factors influencing diagnostic intervals. Legend: Reverse Kaplan-Meier-curves illustrate factors, that significantly contribute to intervals from symptom onset to NL diagnosis on univariate analysis. **(A)** Secondary NL, **(B)** rapidly progressive, asymmetric neuropathy, **(C)** upfront evaluation with either MRI or FDG-PET as opposed to conventional CT, **(D)** and absence of nerve root lesions were associated with shorter intervals from symptom onset to diagnosis

Definitive NL was diagnosed a median of 3 months (range: 1–12 months) after symptom onset (Fig. 4). Demographic, clinical, neuroradiological and pathological data were evaluated to identify prognosticators of diagnostic intervals. All variables assessed are summarized in Supplementary Table 1. Compared to primary NL, secondary disease was diagnosed earlier, i.e. after shorter intervals from neurological symptom-onset (median 2 vs. 6 months; ratio 0.33 (95% confidence interval (95CI): 0.14–0.82; *p* = 0.02; Fig. 5A). The typical clinical presentation of NL, namely asymmetrical and rapidly progressive neuropathy, was associated with an earlier diagnosis as opposed to other clinical manifestations (median 2 vs. 6 months; ratio: 0.33, 95CI: 0.14–0.78; *p* < 0.001, Fig. 5B). Initial evaluation with conventional CT in contrast to other neuroimaging modalities resulted in diagnostic delays (median 5 vs. 2 months; ratio: 2.50, 95CI: 0.98–6.39; *p* = 0.04, Fig. 5C). Among disease locations, NL with involvement of nerve roots were diagnosed after NL localized elsewhere in the PNS (median 4 vs. 1.5 months; ratio: 2.66, 95CI: 1.04–6.81; *p* = 0.049, Fig. 5D). Although cohort size did not permit multivariate analysis, it is noteworthy that patients with primary NL (5/7, 71% vs. 1/15, 7%; *p* = 0.004) were more frequently evaluated with upfront conventional CT. Clinical presentation and course or location of NL lesions were not statistically different between primary and secondary NL cases.

## Discussion

In this study, we present a comprehensive single-institution assessment of the diagnostic work-up in NL encompassing cases diagnosed over a 20-year period at a tertiary referral center. To our knowledge, previous NL studies did not report intervals from symptom onset to NL diagnosis and factors influencing them [2, 11]. Assessing a broad range of variables including clinical, radiological, and pathological parameters, we identified prognosticators of longer intervals from symptom onset to diagnosis. Results may guide physicians in appropriate diagnostic work-up and early diagnosis of NL.

Considering neurolymphomatosis in the differential diagnosis mandates a high degree of suspicion given its rarity. Although our search strategy, did not allow to determine the incidence of neurolymphomatosis at our center, it is estimated to represent < 1% of NHL. Approximately one newly-diagnosed case per year was identified in this study. This is in agreement with previous work by the IPCG, where an average 0.26 cases were diagnosed per year and involved center [2].

Diagnosis remains challenging as integration of clinical, neuroimaging and histopathological data is warranted. Of note, NL diagnosis was reached in all cases from this series *ante mortem*. This observation contrasts earlier studies of NL, where a substantial number of cases was diagnosed *post mortem* only [1]. In an early case study including NL diagnosed between 1972 and 2000, diagnosis was only established by autopsy in 46% of patients [1]. This change is probably owed to improved neuroimaging, that nowadays relies on FDG-PET or MRI rather than conventional CT [2, 3]. In line with this notion, all NL except a single case from our study were at least evaluated with either MRI or FDG-PET. Sensitivity of various neuroradiological modalities in our cohort was similar to previous studies, where FDG-PET carried the highest diagnostic yield (84%) [2]. This underlines FDG-PET as the gold standard neuroimaging technique for staging of FDG-avid NHL within and outside the nervous system. Its use is recommended in recent consensus statements developed by the international conference on malignant lymphomas imaging working group [12, 13]. However, MRI also carried a high sensitivity, is rapidly-available and may be superior for differentiating between other causes of peripheral neuropathy. PNS biopsy also carried a high diagnostic yield (100%), and re-sampling was not necessary in our cohort. The lack of negative biopsies in contrast to previous studies, could be explained by the fact that PNS biopsy sites are chosen based on neuroimaging abnormalities at our center [2]. Other institutions reported sampling peripheral sensory nerves (e.g. sural) irrespective of neuroimaging findings as part of neuropathy work-up, because they carry a lower risk for relevant surgical complications [14, 15].

Median interval from symptom onset to NL diagnosis was 3 months with some cases only diagnosed 12 months after symptom onset. Of note, previously reported diagnostic delays in CNS lymphoma, another rare NHL subtype, resemble NL. A Scandinavian study found CNS lymphoma was diagnosed a median 3 months after symptom-onset [16]. A more recent multi-center study from Spain found diagnosis was made a median 2 months after symptom onset [17]. These intervals are similar to systemic NHL, which was diagnosed after a median of 51 days based on a British study, and substantially longer than in other malignancies such as lung or breast cancer [18].

This study found patients with secondary compared to primary NL and typical (asymmetrical, rapidly-progressive neuropathy) as opposed to atypical presentation were diagnosed earlier. Initial evaluation with conventional CT compared to other imaging modalities and the presence of nerve root lesions were associated with diagnostic delays.

Clinical presentation in primary NL can mimic a wide range of mostly non-malignant conditions, including metabolic (e.g., diabetic neuropathy), autoimmune (e.g., chronic inflammatory demyelinating neuropathy), toxic (e.g., alcohol), hereditary (e.g., hereditary motor sensory neuropathies) or infectious neuropathies (e.g., Lyme disease) [6, 7, 19]. In contrast to most these other etiologies, NL is exceedingly rare. Most patients presenting with signs of neuropathy initially consult a general practitioner or general neurologist, who may not immediately suspect NL, resulting in an increased number of consultations prior to diagnosis [20]. In contrast to primary disease, secondary NL patients are routinely monitored by an interdisciplinary team of oncologists and neuro-oncologists, who may be more inclined to consider NL [21]. Additionally, these patients undergo whole-body imaging for staging as part of their routine follow-up [10]. One case of secondary NL from this study represented an incidental finding on follow-up FDG-PET likely allowing for disease detection before symptoms arose.

Given the broad range of differential diagnoses, NL cases with atypical clinical presentation (e.g., slowly-progressive course or symmetrical distribution) were subject to diagnostic delays. Likely many of these patients were initially mistaken for more common types of peripheral neuropathy such as diabetic neuropathy [22]. Diabetic neuropathy typically presents as a distal symmetric neuropathy with slow progression of symptoms and is among the lead causes of neuropathy in the Western population [23]. Additionally, rapidly progressive symptoms, typically seen in NL, will likely translate into earlier consultation of specialists and definitive diagnosis.

Diagnostic delays in patients initially evaluated with conventional CT may underline its comparatively low sensitivity for NL diagnosis, which was again confirmed in our cohort. Of note, upfront conventional CT was more frequently performed in patients eventually diagnosed with primary NL, in whom the diagnosis may not have been immediately suspected. It may also have been ordered for rapid radiological evaluation of symptomatic patients and negative results may have further delayed appropriate diagnostic work-up. Interestingly, patients with nerve root involvement were also subject to diagnostic delays. Given predominantly lumbosacral nerve roots were infiltrated by NL, findings may have been initially misattributed to radiculopathy from herniated discs, which carries a prevalence of 6% and 43% in the asymptomatic and symptomatic population, respectively [24].

Collectively, NL must be considered in the differential diagnosis in patients presenting with asymmetric, rapidly-progressive neuropathy and timely neuroimaging should be ordered. However, this typical clinical manifestation was only found in half of our patients and hence, other clinical presentations do not rule out NL diagnosis. It should therefore also be considered in all cases of progressive neuropathy that cannot be attributed to an alternative cause or unexpectantly fail treatment. Similarly, non-Hodgkin lymphoma patients who develop neuropathy should be evaluated for NL.

Our study is limited by the small cohort size, its heterogeneity, and the single-center, retrospective design subjecting it to selection bias and precluding multivariate analysis. However, the size of the reported cohort was larger than in most previous studies and results from NL rarity. The largest previous retrospective study reported 50 NL cases diagnosed at 12 institutions from 1993 to 2008 but did not address intervals from symptom onset to diagnosis [2]. Notes from external encounters prior to admission at our center and diagnoses suspected prior to neuroimaging and histopathological evaluation were not available for review. Neurological symptoms and their severity as well as their assessment may be subjective. However, board-certified neurologists, who subspecialized in neuro-oncology, examined all cases and confirmed patient-reported symptoms. Strengths of this study include the comprehensive summary of diagnostic work-up in NL at a tertiary referral center over two decades and the identification of parameters associated with longer diagnostic intervals. Our work may guide initiation of high-yield diagnostic studies in NL, which may expedite NL diagnosis.

Future directions include the development and evaluation of novel diagnostic tools, which may ease and accelerate NL diagnosis. Novel MR-based imaging sequences including diffusion-weighted imaging or spectroscopy, which were not available for our analysis in sufficient case numbers, should be further evaluated and may aid radiological diagnosis. Comparable to NHL within the CNS, liquid biopsy using CSF or serum samples could play a central role during work-up in the near future [25–28]. It may allow to defer PNS biopsy potentially associated with substantial morbidity. Specifically, liquid biopsies detecting MYD88 hotspot variants (p.L265P) have shown promise for the non-invasive diagnosis of CNS lymphoma [25, 26]. However, the genetic landscape of NL has not been comprehensively characterized and it hence remains unclear whether these approaches, successfully used for CNS lymphoma, can be transferred to NL. Genomic characterization of NL tumor specimens may be equally promising as in CNS lymphoma for which distinct genetic alterations have been identified predisposing to nervous system dissemination [29, 30]. Genetic analyses may not only pave the way for liquid biopsy but enhance our pathogenetic understanding of this rare entity potentially allowing for targeted therapy as available for primary CNS lymphomas [31–33].

## Conclusions

In this study, we present a comprehensive single-institution analysis of the diagnostic work-up in NL encompassing 22 cases diagnosed over a 20-year period. Neuroimaging, particularly FDG-PET, constitutes a pillar of modern era NL diagnosis. FDG-PET guided selection of biopsy sites resulted in high diagnostic yields. However, NL diagnosis remains subject to relevant diagnostic delays with some cases from this cohort diagnosed only twelve months after symptom onset. NL type and localization, neuropathy course and distribution, and imaging modality selected for initial evaluation influence diagnostic intervals. Our work may guide prompt initiation of diagnostic work-up in NL, which may expedite NL diagnosis.

## Electronic supplementary material

Below is the link to the electronic supplementary material.

Supplementary Material 1

## Data Availability

Data is provided within the manuscript or supplementary information files.
