# Supplementary material for: Factors influencing timely diagnosis in neurolymphomatosis
Source: J Neurooncol. 2024 Aug 8;170(2):309–17. doi: 10.1007/s11060-024-04792-2 (PMC12799673; doi:10.1007/s11060-024-04792-2)
Supplement: Supplementary file 1 — Supplementary Material 1 [file 11060_2024_4792_MOESM1_ESM.docx]

**Supplementary Figure 1 - Title: NHL characteristics at initial diagnosis in secondary NL**

**
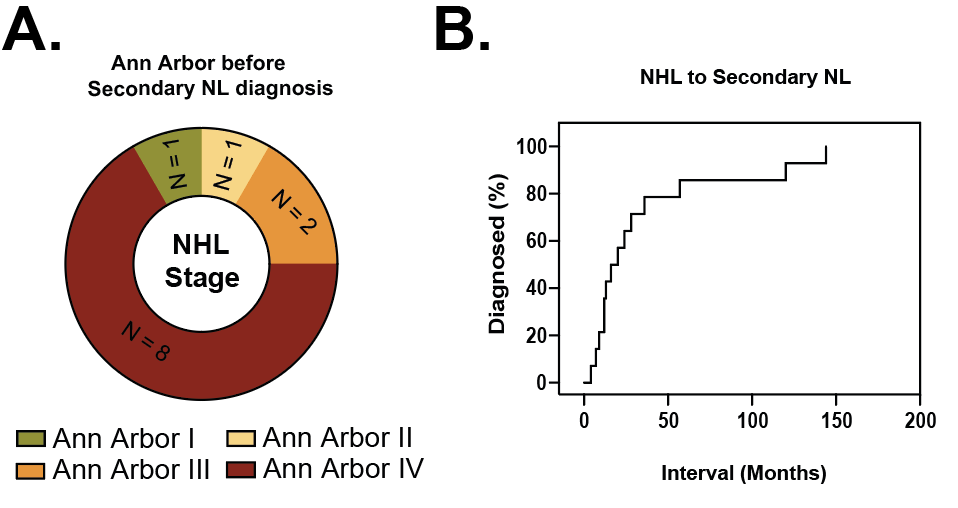
**

**Legend: A.** NHL Ann Arbor stage at initial diagnosis prior to NL is shown for secondary NL. Secondary NL was found in patients previously diagnosed with advanced-stage NHL, corresponding to Ann Arbor stages III and IV. **B.** Reverse Kaplan-Meier-curve depicts intervals from initial NHL to PNS dissemination (secondary NL). Secondary NL was diagnosed a median of 16 months after initial NHL.

**Supplementary Table 1**

**Title:** Univariate Analysis – Parameters influencing diagnostic delays

| Category | Variables |  | Median diagnostic interval (mo) | Ratio (95 CI) | Log-rank test,  P value |
| --- | --- | --- | --- | --- | --- |
| Epidemiology | Gender | Male | 3 | 1.00 (0.42-2.38) | 0.91 |
|  |  | Female | 3 |  |  |
|  | Age group (y) | < Median | 4 | 1.33 (0.58-3.09) | 0.94 |
|  |  | ≥ Median | 3 |  |  |
| Clinical | NL type | Primary | 6 | 3 (1.22-7.36) | 0.02 * |
|  |  | Secondary | 2 |  |  |
|  | Performance status | ECOG < 2 | 3 | 1.00 (0.42-2.38) | 0.85 |
|  |  | ECOG ≥ 2 | 3 |  |  |
|  | Presentation category | Painful polyneuropathy | 3 | 1.00 (0.41-2.45) | 0.67 |
|  |  | Others | 3 |  |  |
|  | Neuropathy course and distribution | Rapidly progressive, asymmetrical | 2 | 0.33 (0.14-0.78) | < 0.001 *** |
|  |  | Others | 6 |  |  |
|  | Location | Peripheral nerve | 2.5 | 0.71 (0.31-1.65) | 0.27 |
|  |  | Others | 3.5 |  |  |
|  |  | Nerve root | 4 | 2.67 (1.04-6.82) | 0.049 * |
|  |  | Others | 1.5 |  |  |
|  |  | Cranial nerve | 2.5 | 0.71 (0.24-2.11) | 0.53 |
|  |  | Others | 3.5 |  |  |
|  | Histopathological characteristics | DLBCL | 4 | 2.00 (0.78-5.11) | 0.07 |
|  |  | Others | 2 |  |  |
|  | Interval from NHL-NL | ≤ Median | 4 | 2.00 (0.70-5.70) | 0.49 |
|  |  | > Median | 2 |  |  |
| Diagnostic modalities | Histopathological diagnosis | Yes | 3 | 0.75 (0.33-1.73) | 0.49 |
|  |  | No | 4 |  |  |
|  | First imaging: CT | Yes | 5 | 2.5 (0.98-6.39) | 0.04 * |
|  |  | No | 2 |  |  |
|  | CSF cells increased | Yes | 2.5 | 0.71 (0.26-1.97) | 0.91 |
|  |  | No | 3.5 |  |  |
|  | CSF protein increased | Yes | 3 | 1.20 (0.39-3.72) | 0.78 |
|  |  | No | 2.5 |  |  |
|  | CSF cytology/FACS | Positive | 2 | 0.57 (0.21-1.57) | 0.84 |
|  |  | Negative | 3.5 |  |  |
| **Abbreviations:** Mo, Months; NL, neurolymphomatosis; ECOG, Eastern Cooperative Oncology Group; DLBCL, diffuse large B-cell lymphoma; NHL, non-Hodgkin lymphoma; CT, computed tomography; MRI, magnetic resonance imaging; FDG-PET, fluorodeoxyglucose – positron emission tomography; CSF, cerebrospinal fluid; *, p < 0.05; **, p < 0.01; *** p < 0.001 | | | | | |
